# Trichostatin A resistance is facilitated by HIF-1α acetylation in HeLa human cervical cancer cells under normoxic conditions

**DOI:** 10.18632/oncotarget.23327

**Published:** 2017-12-16

**Authors:** Jae-Wook Lee, Dong Hee Yang, Sojin Park, Hae-Kyoung Han, Jong-Wan Park, Bo Yeon Kim, Sung Hee Um, Eun-Yi Moon

**Affiliations:** ^1^ Department of Bioscience and Biotechnology, Sejong University, Seoul 05006, South Korea; ^2^ Department of Pharmacology, College of Medicine, Seoul National University, Seoul 03080, South Korea; ^3^ World Class Institute, Anticancer Agents Research Center, Korea Research Institute of Bioscience and Biotechnology, Ochang, Cheongwon 28116, South Korea; ^4^ Department of Molecular Cell Biology, Samsung Biomedical Research Institute, Sungkyunkwan University School of Medicine, Suwon, Gyeonggi-do 16419, South Korea; ^5^ Department of Health Sciences and Technology, SAIHST, Samsung Medical Center, Sungkyunkwan University, Seoul 06351, South Korea

**Keywords:** trichostatin A, HIF-1α, acetylation, drug-resistance, normoxic conditions

## Abstract

Trichostatin A (TSA) is an anticancer drug that inhibits histone deacetylases (HDACs). Hypoxia-inducible factor 1 (HIF-1) participates in tumor angiogenesis by upregulating target genes, such as vascular endothelial growth factor (VEGF). In the present study, we investigated whether TSA treatment increases HIF-1α stabilization *via* acetylation under normoxic conditions, which would lead to VEGF upregulation and resistance to anticancer drugs. TSA enhanced total HIF-1α and VEGF-HRE reporter activity under normoxic conditions. When cells were transfected with GFP-HIF-1α, treatment with TSA increased the number of green fluorescence protein (GFP)-positive cells. TSA also enhanced the nuclear translocation of HIF-1α protein, as assessed by immunoblotting and as evidenced by increased nuclear localization of GFP-HIF-1α. An increase in the interaction between HIF-1α and the VEGF promoter, which was assessed by a chromatin immunoprecipitation (ChIP) assay, led to activation of the VEGF promoter. TSA acetylated HIF-1α at lysine (K) 674, which led to an increase in TSA-induced VEGF-HRE reporter activity. In addition, TSA-mediated cell death was reduced by the overexpression of HIF-1α but it was rescued by transfection with a HIF-1α mutant (K674R). These data demonstrate that HIF-1α may be stabilized and translocated into the nucleus for the activation of VEGF promoter by TSA-mediated acetylation at K674 under normoxic conditions. These findings suggest that HIF-1α acetylation may lead to resistance to anticancer therapeutics, such as HDAC inhibitors, including TSA.

## INTRODUCTION

The survival rates of tumor patients are decreasing because of tumor cell resistance to anticancer chemotherapies [1]. This is due to acute alterations in the tissues surrounding the tumor and in the environment as a result of treatment with anticancer therapeutics, which facilitate the drug resistance of tumor cells.

As many types of solid tumors adaptively respond to hypoxic (low O_2_ levels) stress, several survival pathways in hypoxic tumor cells are activated to carry out those essential biological processes that differ from the processes of normal cells [2]. Hypoxia-inducible factor 1 (HIF-1) is an oxygen-sensitive transcription factor [3] comprised of α and β subunits that form a heterodimer that binds to the hypoxia response element (HRE) region in DNA [3–5]. HIF-1α controls the expression of downstream target genes, including glucose transporter 1 (GLUT1), vascular endothelial growth factor (VEGF), insulin growth factor 2 (IGF2), transforming growth factor (TGF), erythropoietin (EPO) and so on. HIF-1α is correlated with vascular density [6, 7], tumor metastasis, angiogenesis, poor patient prognosis, as well as anticancer drug resistance [2]. Under normoxic conditions, HIF-1α hydroxylation of proline residues within its oxygen-dependent degradation (ODD) domain allows for HIF-1α association with von Hippel–Lindau (VHL) protein. Then, HIF-1α is degraded by 26S proteasome [8, 9]. Additionally, anticancer efficacy is reduced by HIF-1α overexpression or hypoxic conditions, which leads to the induction of drug resistance [10, 11]. Tumor hypoxia also leads to resistance to radiotherapy [12]. However, it is unknown whether HIF-1α can induce drug resistance of histone deacetylase (HDAC) inhibitor trichostatin A (TSA).

TSA was first obtained from *Streptomyces hygroscopicus* as an antifungal antibiotic that is active against *Trichophyton* species and is used for the specific inhibition of HDACs including the class I and II, but not class III HDACs [13]. Highly acetylated histones are accumulated by TSA [14]. Reduced HDAC activity blocks the cell cycle, cell proliferation, and apoptosis [15]. TSA inhibits the hypoxia-induced accumulation of HIF-1α and VEGF under hypoxic conditions [16–19]. TSA also decreases HIF-1αlpha protein levels and VEGF expression in multiple cancer cells, including HeLa cells [20–23]. In contrast, changes in various intracellular molecules play a role in drug resistance. For example, overexpression of multidrug resistance-associated protein 8 (MRP8) [24], glucose-regulated protein 78 kDa (GRP78/BiP) [25], or p21WAF1 [26] leads to resistance to HDAC inhibitor-induced cancer cell apoptosis. However, it is unknown whether drug resistance can be induced by treatment with antitumor therapeutics, such as the HDAC inhibitor TSA, *via* alterations in HIF-1α acetylation under normoxic conditions.

We determined whether HIF-1α acetylation by TSA affects tumor cell survival *via* nuclear translocation and binding to the HRE of the VEGF promoter. Our results suggest that the therapeutic effects of anticancer agents such as TSA may be hampered by HIF-1α acetylation under normoxic conditions.

## RESULTS

### TSA enhanced VEGF-HRE reporter activity and HIF-1α expression

**Figure 1:**
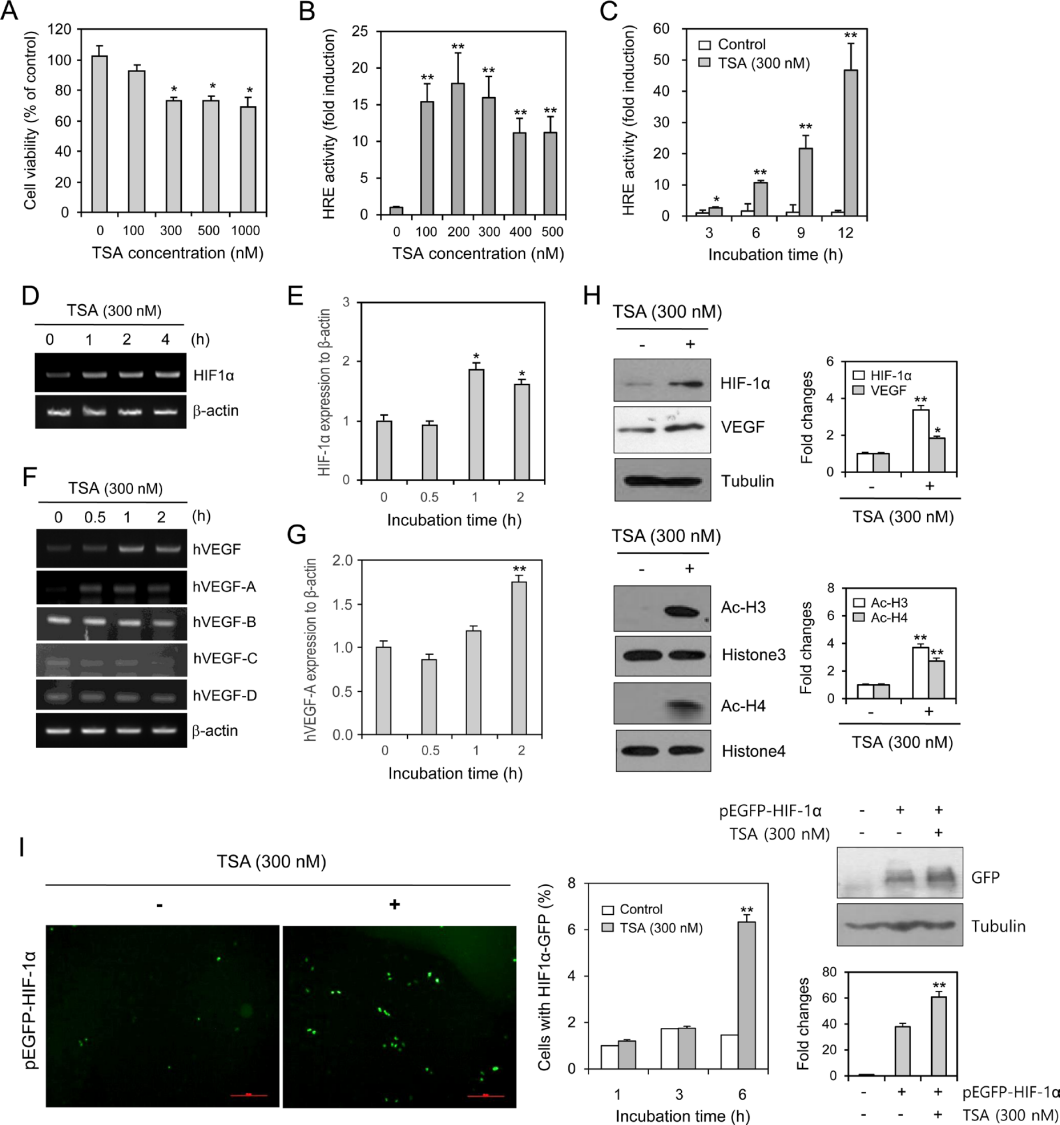
TSA enhanced VEGF-HRE reporter activity and the amount of HIF-1α protein HeLa cells were incubated with various concentrations of TSA for 48 h. Cell viability was measured by MTT assay (**A**). HeLa cells were transfected with VEGF-HRE-pSV40min and incubated with various concentrations of TSA including 300 nM (**B**) or with 300 nM TSA for various times (**C**). VEGF-HRE activity was measured by using luminometer (B and C). HeLa cells were treated with 300 nM TSA for various times (**D**–**H**). HIF-1α or VEGF expression was measured with RT-PCR (D and F) or realtime Q-PCR (E and G). Western blot analysis was performed for the detection of HIF-1α, VEGF (H, top), histone 3/4 and acetylated histone 3/4 (H, bottom). Each band was quantified by using IamgeJ 1.34 and the results were represented as fold changes to control. (H, top and bottom right). HeLa cells were transfected with pEGFP-C3-HIF-1α plasmid and incubated with 300 nM TSA. GFP was observed under fluorescence microscope with 200× magnification (**I**, left). Then, the number of cells with GFP-HIF-1α expression was counted and represented as bar graph (I, middle). GFP expression was detected with western blot analysis (I, right top). Each band was quantified by using IamgeJ 1.34 and the results were represented as fold changes to control. (I, right bottom). Data are the representative of three experiments. Data in bar graph represent mean ± SD. ^*^p < 0.05; ^**^p < 0.01, significantly different from TSA-untreated control.

To examine the effects of TSA on cell viability, HeLa cells were treated with TSA for 48 h. TSA treatment decreased cell viability at concentrations ranging from 300 nM to 1,000 nM, as determined by the MTT assay (Figure 1A). TSA also increased VEGF-HRE reporter activity (Figure 1B and 1C). The mRNA expression levels of HIF-1α (Figure 1D and 1E), total VEGF, and VEGF-A (Figure 1F and 1G) were enhanced by TSA treatment. No changes were detected in VEGF-B, VEGF-C, or VEGF-D (Figure 1F). TSA treatment elevated the protein levels of HIF-1α and VEGF (Figure 1H, top). HDAC inhibition by TSA was confirmed by an increase in acetylation at histones 3 and 4 (Figure 1H, bottom). Transfection with pEGFP-HIF-1α caused an increased number of TSA-treated cells expressing GFP-HIF-1α (Figure 1I, left and middle). HIF-1α expression was also increased by TSA treatment, which was detected by western blot analysis (Figure 1I, right). These data suggest that an increase in VEGF-HRE reporter activity by TSA might be associated with the binding of HIF-1α to the HRE following nuclear localization of HIF-1α under normoxic conditions.

### VEGF-HRE reporter activity in various types of cells was augmented by TSA treatment

**Figure 2:**
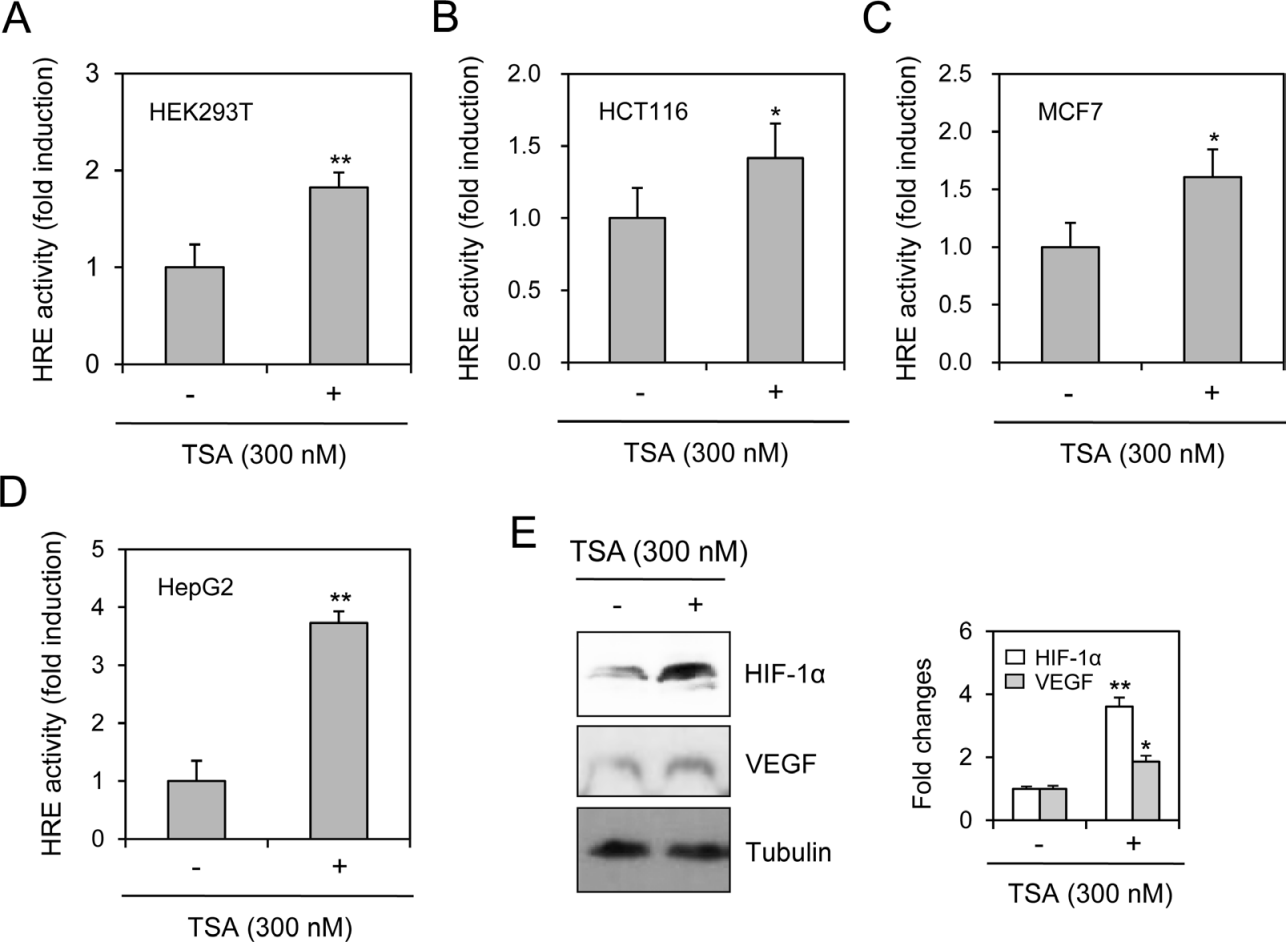
TSA treatment increased VEGF-HRE reporter activity in various type of cells VEGF-HRE-pSV40min plasmids were transfected into HEK293T human embryonic kidney cells (**A**), HCT116 human colorectal carcinoma cells (**B**), MCF7 human breast adenocarcinoma cells (**C**) and HepG2 human hepatocellular carcinoma cells (**D**). Each cell was incubated in the presence or absence of 300 nM TSA for 12 h. VEGF-HRE activity was measured by using luminometer (A–D). HepG2 cells were incubated with 300 nM TSA and cell lysates were prepared. Western blot analysis was performed for the detection of HIF-1α and VEGF (**E**, left). Each band was quantified by using IamgeJ 1.34 and the results were represented as fold changes to control. (E, right). Data are the representative of three experiments. Data in bar graph represent mean ± SD. ^*^p < 0.05; ^**^p < 0.01, significantly different from TSA-untreated control.

We assessed the effects of TSA on VEGF-HRE reporter activity in various types of cells. Our data showed that VEGF-HRE activity was increased by the incubation with TSA in HEK293T human embryonic kidney cells (Figure 2A), HCT116 human colorectal carcinoma cells (Figure 2B), MCF7 human breast adenocarcinoma cells (Figure 2C) and HepG2 human hepatocellular carcinoma cells (Figure 2D). TSA-induced increase in VEGF-HRE activity was the highest in HepG2 cells under our experimental condition. Then, we tested the expression of HIF-1α and VEGF in HepG2 cells. As shown in Figure 2E, HIF-1α and VEGF expression in HepG2 cells was increased by TSA treatment under normoxic conditions. It suggests that TSA might enhance VEGF-HRE reporter activity via nuclear localization of HIF-1α and the binding of HIF-1α to the HRE in various types of cells including HeLa cells.

### TSA treatment increased the nuclear localization of HIF-1α

**Figure 3:**
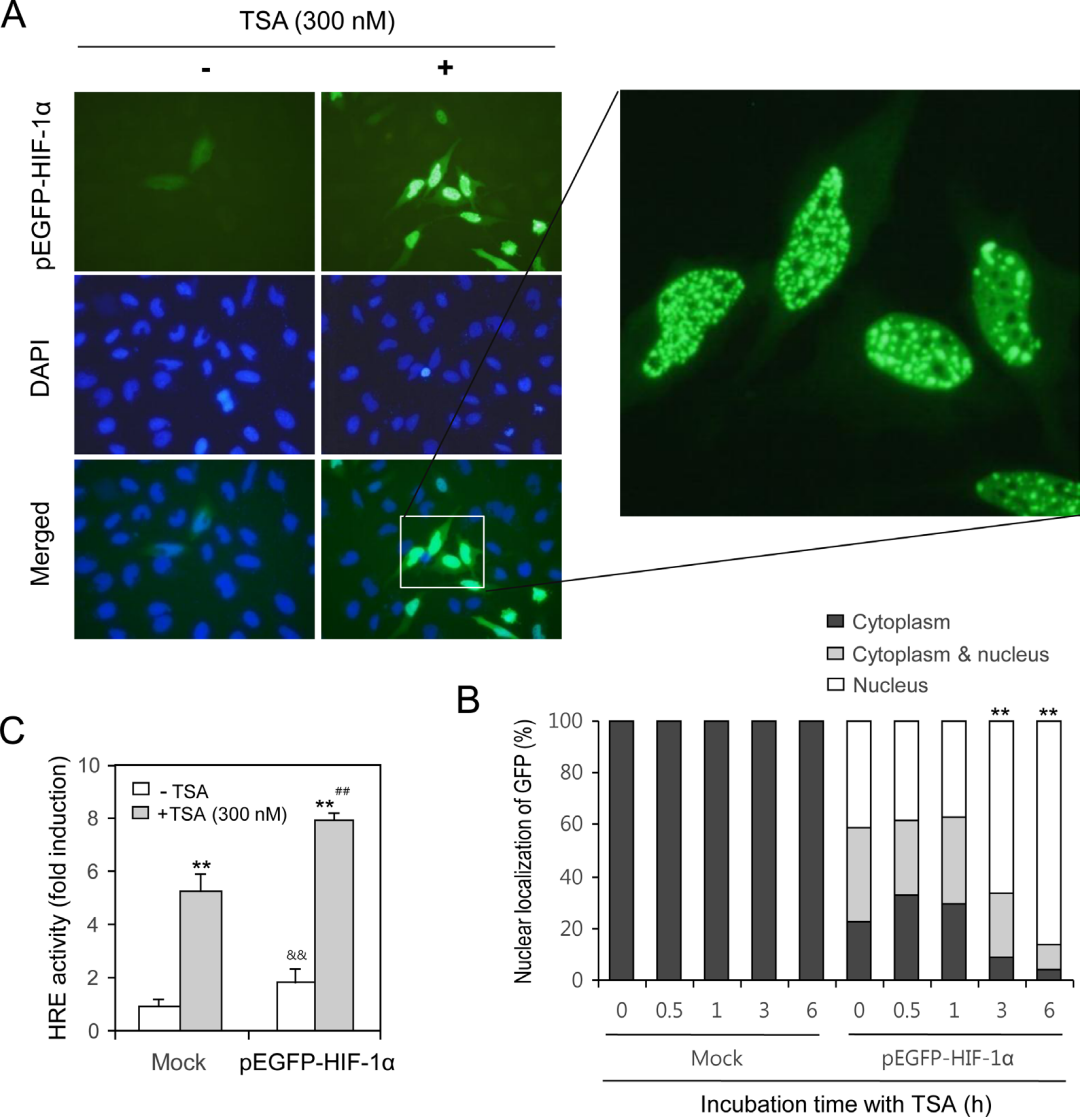

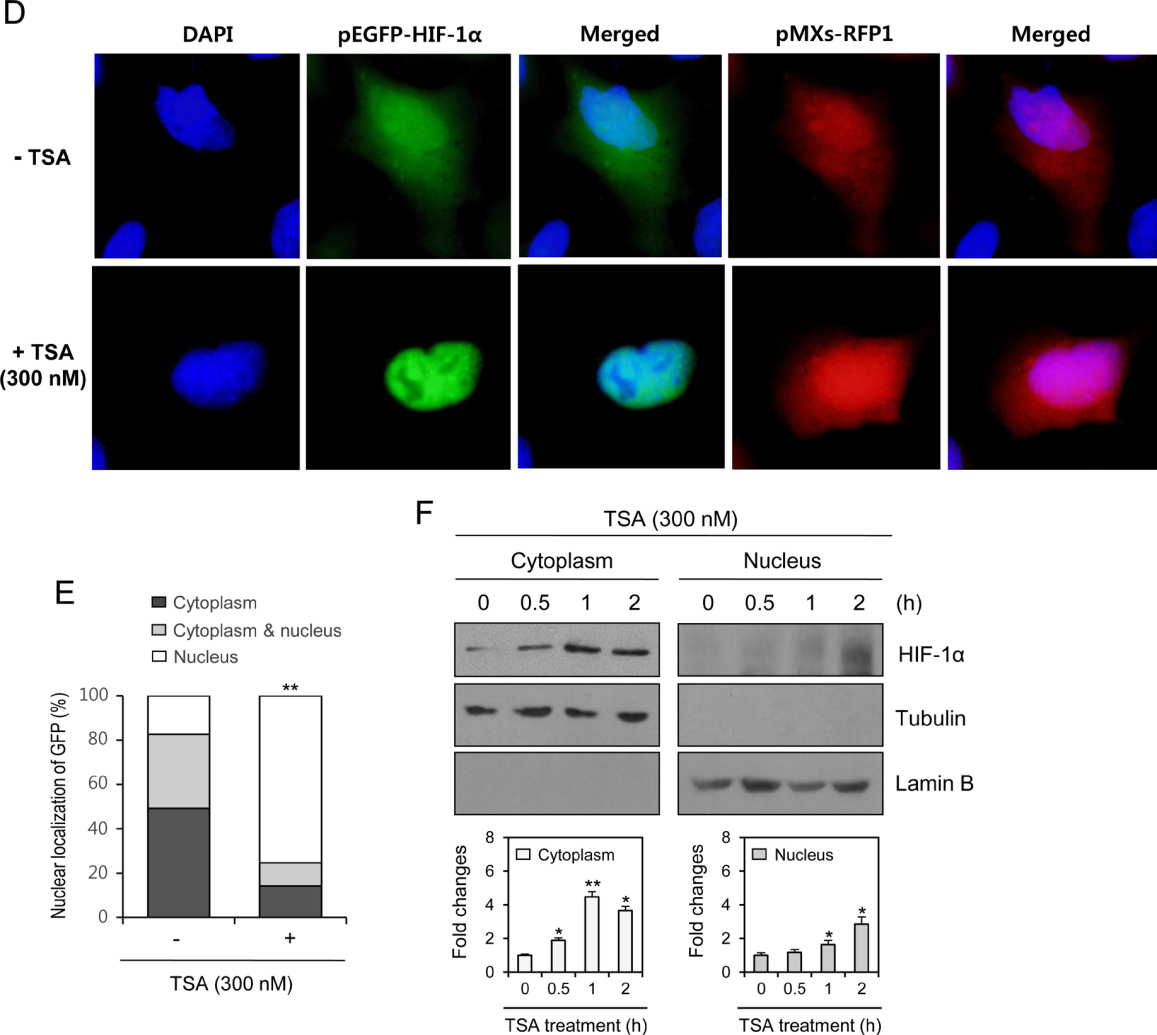
TSA treatment increased nuclear localization of HIF-1α HeLa cells were transfected with pEGFP-C3-HIF-1α plasmid and incubated with 300 nM TSA. GFP expression in cytoplasm and/or nucleus was observed under fluorescence microscope with 400× (**A**) or 1,000× (A, inset) magnification. Three blinded individuals counted the percentage of cells with the nuclear translocation of GFP-HIF-1α under fluorescence microscope (**B**). HeLa cells were co-transfected with VEGF-HRE-pSV40min and pEGFP-HIF-1α plasmids. Then, cells were incubated with 300 nM TSA and VEGF-HRE activity was measured by using luminometer (**C**). HeLa cells were co-transfected with pMXs-RFP1 and pEGFP-C3-HIF-1α plasmid and incubated with 300 nM TSA. GFP expression in cytoplasm and/or nucleus with RFP expression in cytoplasm was observed under fluorescence microscope (**D**). Three blinded individuals counted the percentage of cells with the nuclear translocation of GFP-HIF-1α (**E**). HeLa cells were incubated with 300 nM TSA and nuclear fraction was separated from cytoplasm. HIF-1α was detected with western blot analysis (E, top). Each band was quantified by using IamgeJ 1.34 and the results were represented as fold changes to control (E, bottom). Data are the representative of three experiments. Data in bar graph represent mean ± SD. ^*^p < 0.05; ^**^p < 0.01, significantly different from TSA-untreated control group transfected with mock or pEGFP-C3-HIF-1α. ^&&^*p* < 0.01, significantly different from mock-transfectd group in the absence of TSA. ^##^*p* < 0.01, significantly different from mock-transfectd group in the presence of TSA.

The effects of TSA-mediated HIF-1α expression on VEGF-HRE were examined by transfecting cells with pEGFP-C3-HIF-1α plasmid and determining the nuclear localization of HIF-1α. As shown in Figure 3A, the nuclear localization of GFP-HIF-1α was increased by TSA treatment. The relative percentage of nuclear localization of GFP was increased in the nucleus 1 h after TSA treatment (Figure 3B). No changes were detected in mock-transfected cells (Figure 3B). Additionally, when cells were co-transfected with pEGFP-C3-HIF-1α and the HRE reporter plasmid, VEGF-HRE reporter activity increased following TSA treatment (Figure 3C). We re-affirmed that TSA enhanced the nuclear localization of HIF-1α by the co-transfection with pMXs-RFP1 and pEGFP-C3-HIF-1α plasmid. HeLa cells were incubated with 300 nM TSA and GFP expression in cytoplasm and/or nucleus with RFP expression in cytoplasm was observed under fluorescence microscope. While GFP fluorescence was shifted from cytoplasm to nucleus, no translocation in RFP was detected (Figure 3D). The percentage of cells with GFP-HIF-1α was increased in nucleus but it was reduced in cytoplasm (Figure 3E). When cells were separated into cytoplasmic and nuclear fractions after TSA treatment, HIF-1α amount was increased in both cytoplasm and nuclear fractions (Figure 3F). It suggests that HIF-1α in cytosol should be primarily increased by TSA treatment and translocated into nucleus, which binds to the VEGF promoter to induce its mRNA transcription.

### TSA treatment increases HRE binding of HIF-1α

**Figure 4:**
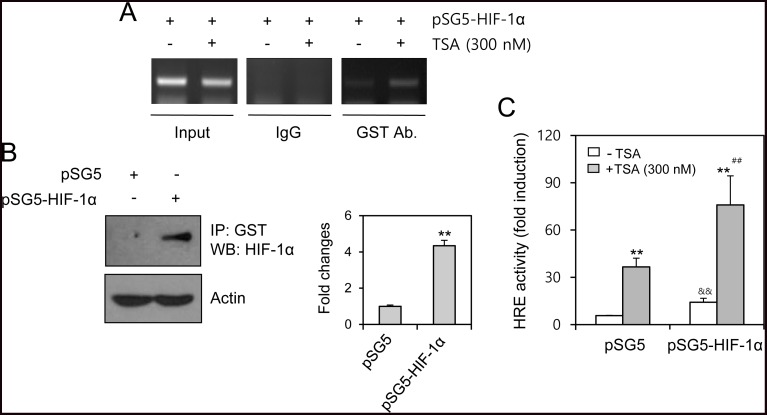
TSA treatment increased HIF-1α binding on HRE of VEGF promoter HeLa cells were transfected with pSG5-HIF-1α, treated with TSA and fixed with 10% formaldehyde. Their chromatin extracts were immunoprecipitated with anti-GST antibodies. DNA fragments were subjected to PCR analysis using primer sets spanning HIF-1α binding site (–1,037 ~ –869 bp) of VEGF gene promoter regions (**A**). Cell lysates were prepared and immunoprecipitated with anti-GST antibodies. HIF-1α was detected with western blot analysis (**B**, left). Each band was quantified by using IamgeJ 1.34 and the results were represented as fold changes to control. (B, right). VEGF-HRE activity was measured by using luminometer. Data are the representative of three experiments. Data in bar graph represent mean ± SD. ^**^p < 0.01, significantly different from TSA-untreated control group transfected with mock or pSG5-HIF-1α. ^&&^*p* < 0.01, significantly different from pSG5 control vector-transfectd group in the absence of TSA. ^##^*p* < 0.01, significantly different from pSG5 control vector-transfectd group in the presence of TSA (**C**).

We next examined the binding of HIF-1α to the VEGF promoter by transfecting pSG5-HIF-1α and performing a chromatin immunoprecipitation (ChIP) assay. TSA treatment enhanced HIF-1α binding to the HRE of hVEGF-A (Figure 4A). HIF-1α amount in cells transfected with pSG5-HIF-1α was confirmed by immunoprecipitation with anti-GST antibodies (Figure 4B). VEGF-HRE reporter activity was also increased by co-transfection with pSG5-HIF-1α (Figure 4C). These results suggest that TSA treatment induces the movement of HIF-1α into the nucleus for binding to the HRE of the VEGF promoter.

### TSA treatment increases the acetylation of HIF-1α at lysine 674

**Figure 5:**
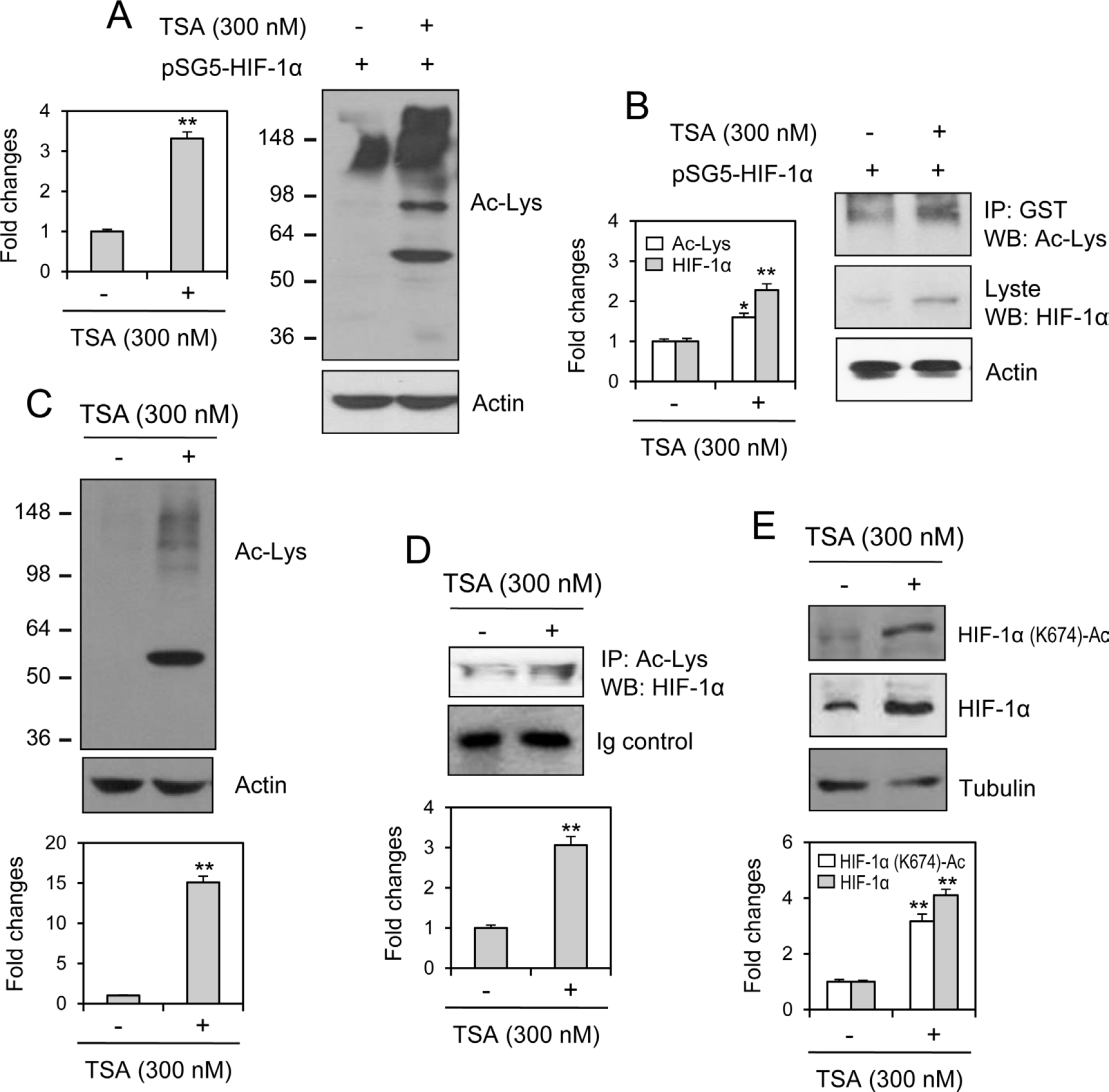
HIF-1α acetylation at lysine 674 was increased by TSA treatment HeLa cells were transfected with pSG5-HIF-1α and cell lysates were prepared. Acetylated lysine was detected by western blot analysis (**A**). Cell lysates were immunoprecipitated with anti-GST antibodies. Acetylated lysine and HIF-1α was detected with western blot analysis (**B**). HeLa cells were incubated with 300 nM TSA and cell lysates were prepared. Acetylated lysine was detected by western blot analysis (**C**). Cell lysates were immunoprecipitated with anti-acetylated lysine antibodies. HIF-1α was detected with western blot analysis (**D**). HIF-1α acetylation at lysine (K) 674 was detected with western blot analysis (**E**). Each band was quantified by using IamgeJ 1.34 and the results were represented as fold changes to control. Data in the bar graph represent the means ± SED. ^*^p < 0.05; ^**^p < 0.01; significant difference as compared to TSA-untreated control (A and B, left, C–E, bottom).

Because TSA is an HDAC inhibitor [13], we determined whether the acetylated lysine (K) is the mechanism underlying the nuclear localization of HIF-1α induced by TSA. When cells were transfected with pSG5-HIF-1α and treated with TSA, acetylation was increased in many proteins (Figure 5A). HIF-1α acetylation was detected by immunoprecipitating GST-HIF-1α and immunoblotting acetylated lysine (Figure 5B). We also determined whether endogenous HIF-1α might be localized into nucleus via lysine acetylation induced by TSA. As shown in Figure 5C, acetylation was increased in many proteins following TSA treatment. HIF-1α acetylation was detected by immunoprecipitating acetylated proteins and immunoblotting HIF-1α (Figure 5D). We also detected TSA-mediated acetylation of HIF-1α at K674 (Figure 5E). Results implicate that antitumor effect of TSA might be modified by the acetylation of HIF-1α at K674.

### Drug resistance to TSA is dependent on HIF-1α acetylation at lysine 674

**Figure 6:**
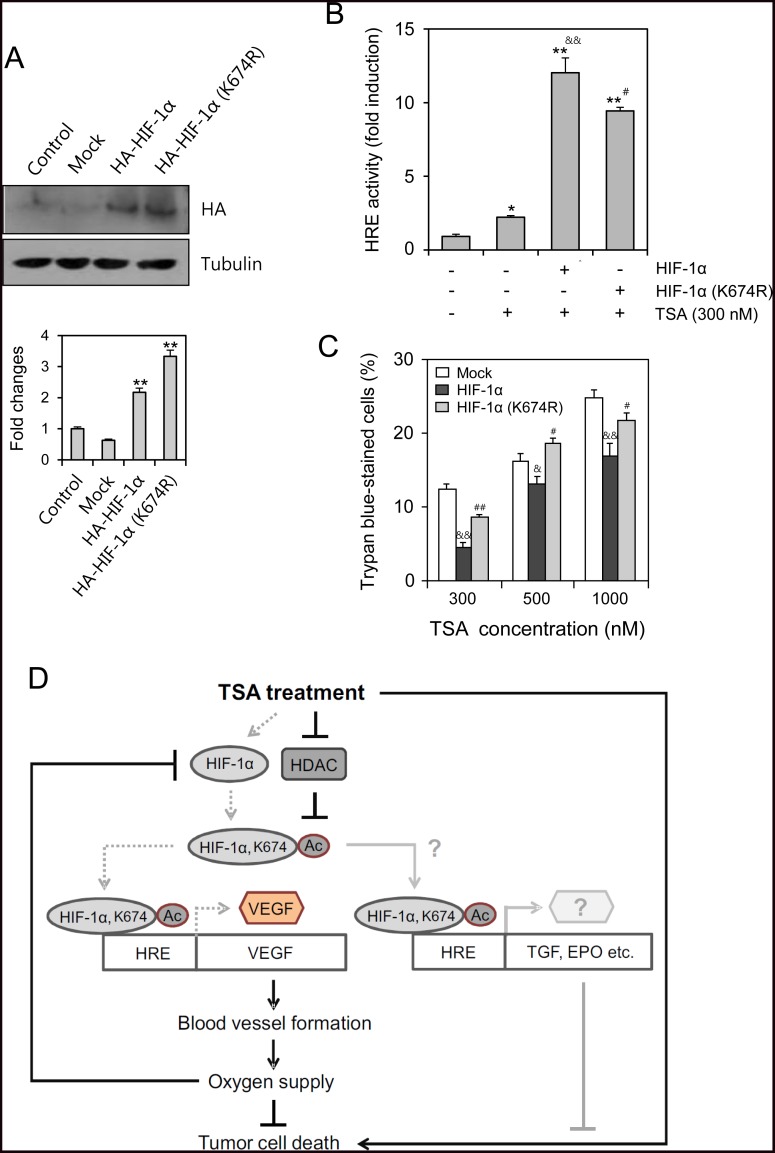
TSA-resistance was dependent on HIF-1α acetylation at lysine (K) 674 HeLa cells were transfected with HA-HIF-1α or HA-HIF-1α K674R in the absence (**A** and **C**) or presence (**B**) of VEGF-HRE promoter. Wildtype or mutant HIF-1α expression was detected with anti-HA antibodies by western blot analysis (A, top). Each band was quantified by using IamgeJ 1.34 and the results were represented as fold changes to control (A, bottom). VEGF-HRE activity was measured by using luminometer (B). Dead cells were detected by trypan blue exclusion assay. Trypan blue-stained cells represent dead cells (C). Data were the representative of three experiments. Data in bar graph represent mean ± SD. ^*^p < 0.05; ^**^p < 0.01, significantly different from control group transfected with mock (A, bottom) or TSA-untreated control group (B). ^&^*p* < 0.05; ^&&^*p* < 0.01, significantly different from mock-transfectd group at each concentration of TSA. ^#^*p* < 0.05; ^##^*p* < 0.01, significantly different from HA-HIF-1α-transfectd group at each concentration of TSA (B and C). This is the scheme for the reduction of tumor cell death by TSA treatment. Our findings are indicated by gray dotted lines that could be additionally explained by the possible pathways represented by gray lines. Black lines are from the results reported already in the literature (**D**).

According to TSA-mediated acetylation of HIF-1α at lysine (K) 674, we examined the changes in antitumor effect of TSA by the mutation at K674. To compare the effect of wildtype and mutant of HIF-1α on TSA-induced cancer cell death, cells were transfected with the wildtype or mutant (K674R) of HIF-1α (Figure 6A). The effect of HIF-1α on HRE was confirmed by the reduction of VEGF-HRE reporter activity in HIF-1α mutant exchanged lysine (K) 674 to arginine (K674R)-transfected group as compared to wildtype HIF-1α-transfected group (Figure 6B). Then, we assessed the changes in antitumor effect by the trypan blue exclusion assay. As shown in Figure 6C, the percentage of trypan blue-stained dead cells was reduced by overexpression of wildtype HIF-1α, which was rescued by the transfection with HIF-1α mutant (K674R). No significant changes were detected in the effect of vehicle on the changes in trypan blue-stained dead cell number. It suggests that the resistance to TSA could be associated with HIF-1α acetylation at K674. We propose the mechanism on TSA resistance in Figure 6D, which might be mediated by the increase in blood vessel formation and oxygen supply of VEGF to tumor cell survival. Additionaly, cell death inhibition by TSA-induced HIF-1α might be associated with various target gene expression such as TGF, EPO and so on to control tumor cell growth and survival.

## DISCUSSION

The efficacy of anticancer therapeutics is limited by tumor cells with resistance to chemotherapy, thus decreasing the survival rate of tumor patients [1]. Among the many significant factors that induce resistance, hypoxic (low O_2_ levels) stress surrounding many types of solid tumor tissue environments activates essential biological responses that differ from those in normal cells [2]. The anticancer efficacy of paclitaxel is reduced by HIF-1α overexpression or hypoxic conditions [10, 11]. The acetylation levels of target proteins may determine the responsiveness or sensitivity of different types of cancer cells to anticancer agents [19]. The expression of GRP78/BiP or MRP8 or p21WAF1 induces drug resistance to HDAC inhibitors, such as TSA [24–26], which accumulates highly acetylated histones [14]. In acute myeloid leukemia (AML) cells, HDAC inhibitors induce drug resistance *via* hyperacetylation of histone proteins in the promoter regions of MDR1, BCRP, and MRP8 [24], indicating that HIF-1α acetylation may also regulate the effects of anticancer therapeutics. However, little is known regarding TSA resistance induced by changes in the level of HIF-1α via acetylation. In the present study, we investigated whether TSA-mediated HIF-1α acetylation affects tumor cell survival *via* nuclear translocation and binding to the HRE of the VEGF promoter using HeLa cells for the continuation of our previous study [10, 27, 44]. Our data showed that TSA increased HRE activity of the VEGF promoter and the amount of HIF-1α protein under normoxic conditions (Figure 1). We confirmed the effect of TSA on VEGF promoter activation using other types of cells including HEK293T human embryonic kidney cells, HCT116 human colorectal carcinoma cells, MCF7 human breast adenocarcinoma cells and HepG2 human hepatocellular carcinomar cells (Figure 2). TSA treatment also increased the nuclear localization of HIF-1α and it's binding to the HRE of the VEGF promoter (Figures 3 and 4). TSA acetylated HIF-1α at K674 (Figure 5), which led to the activation of VEGF-HRE activity and the inhibition of tumor cell death (Figure 6). These data suggest that drug resistance to TSA may be dependent on HIF-1α acetylation at K674.

While the percentage of GFP-HIF-1α-positive cells was increased at 3 and 6 h after TSA treatment (Figure 3B), VEGF-HRE activity was increased from 1 to 12 h (Figure 1C). VEGF-A expression by RT-PCR was increased from 0.5 to 2 h after TSA treatment (Figure 1F), which is not consistent with data increased at 2 h after TSA treatment by Q-PCR (Figure 1G). Then, we confirmed nuclear translocation of HIF-1α at common effective time, 2 h after TSA treatment by western blot analysis (Figure 3F). It suggests that nuclear translocation of HIF-1α might be gradually increased from 1 to 6h after TSA treatment and TSA-mediated HIF-1α could be also degraded at the same time under normoxic condition.

TSA is a natural product that inhibits HDACs of Class I (HDAC1, 2, 3, and 8) and Class II (HDAC4, 5, 6, 7, 9, and 10) [14, 28, 29]. TSA inhibited proliferation of eight breast carcinoma cell lines with mean ± SD IC_50_ of 124.4 ± 120.4 nM (range, 26.4–308.1 nM) *in vitro*. TSA had also pronounced antitumor activity *in vivo* when administered to rats at a dose of 0.5 mg/kg (≒1,650 nM) by daily injection for 4 weeks compared with control animals. Furthermore, TSA did not cause any measurable toxicity in doses of up to 5 mg/kg (≒16,500 nM) [30]. Due to the direct contact of TSA to tumor cells *in vitro*, we tested the effect of TSA on HeLa cells from 100 nM to 1,000 nM for 48 h-incubation (Figure 1). So, we thought that these concentrations are physiologically or clinically reasonable to see whether tumor cell death might be modified by TSA treatment.

In the beginning of our study, we tested the effect of TSA as one of anticancer agents on HeLa cell death. As shown in Figure 1A, when tumor cells were incubated with TSA for 48 h, we observed that tumor cell death was significantly increased from 300 nM which is the lowest concentration under our experimental condition. TSA-induced tumor cell death was time-dependently increased and little changes were detected by the incubation with 300 nM TSA for less than 12 h [27]. Then, we are interesting to see what is happened in tumor cell death by the treatment with 300 nM or less concentration of TSA for most experiments in our study.

Anticancer efficacy is reduced by HIF-1α overexpression or hypoxic conditions, which can lead to drug resistance [10–12]. It is also possible for drug resistance to be induced by HIF-1α acetylation. HDAC4 regulates HIF-1α acetylation, which can be increased by HDAC4 short hairpin RNA (shRNA) and decreased by HDAC4 overexpression. In contrast, HIF-1α protein stability is inhibited by HDAC4 shRNA, which leads to a decrease in both the HIF-1α transcriptional activity and target gene expression [17]. Whereas no changes were shown in HIF-1α stability by HDAC1 or HDAC3 shRNA [17], HDAC1 and HDAC3 positively regulate HIF-1α stability *via* directly binding to the ODD domain of HIF-1α [21]. HDAC1 also regulates VEGF expression in normal keratinocytes (HaCaT cells) [31]. Endogenous HIF-1α increase by TSA treatment could hamper antitumor effect of TSA, which might be resulted in the reduction of TSA-mediated cell death. Our results showed that TSA increased the number of GFP-HIF-1α-positive cells in Figure 1I, which might be associated with the acetylation of K674 in HIF-1α (Figure 5). VEGF-HRE activity was much more enhanced by HIF-1α overexpression (Figure 6B), which was inhibited by mutant HIF-1α (K674R) overexpression. This is why the percentage of tumor cell death by TSA treatment looks like decreased by HIF-1α overexpression, which was overcome by mutant HIF-1α (K674R) overexpression (Figure 6C). So, antitumor effect of TSA might be increased by the inhibition of endogenous HIF-1α level. It implicates that GFP-HIF-1α-positive cells are more resistant to TSA-mediated cell death. These results demonstrate that drug resistance could be regulated by K674 acetylation in HIF-1α by various types of HDAC inhibitors, such as TSA.

It has been reported that silent mating type information regulation 2 homolog 1 (SIRT1), NAD-dependent deacetylase, binds to HIF-1α and deacetylates it at K674 [32]. So, it can't be ruled out the possibility that TSA might inhibit SIRT1 to increase the acetylation of K674 in HIF-1α. Except the acetylation of K674 in HIF-1α, K532 is another acetylation site. ARD1 protein specifically acetylates K532 in the oxygen-dependent degradation (ODD) domain of HIF-1 residues 401–603, which regulates a HIF-1α stability [33]. K532 acetylation in HIF-1α increases the stability of Von Hippel-Landau (VHL) binding to HIF-1α and leads to the rapid proteasomal degradation of HIF-1α via ubiquitination [33]. Therefore, it will be further studied whether TSA might inhibit ARD1 and prevent VHL binding to and the degradation of HIF-1α through K532 acetylation.

VEGF is essential for the blood vessel formation to maintain oxygen supply. HIF-1α increased by TSA-mediated acetylation enhances VEGF expression with HRE, which might be the effort for tumor cells to be survived in the environment with cell death signal. So, the increase in VEGF expression by TSA-induced HIF-1α acetylation could be resulted in promoting blood vessel formation and oxygen supply to increase tumor cell survival. Then, tumor cell death could be decreased by the increase in VEGF expression, which leads to TSA resistance. In the meanwhile, VEGF-HRE activity (~ 9 fold changes) by HIF-1α (K674R) lacking the acetylation site was decreased roughly 28% as compared to its activity (~ 12.5 fold changes) by wildtype HIF-1α (Figure 6B). This is rather smaller than VEGF-HRE activity (~ 45 fold changes) in Figure 1. It might be due to the overexpression of these HIF-1α genes in the cell (Figure 6B). So, the effect of acetylation by TSA on VEGF-HRE activity is much stronger on just endogenous HIF-1α gene.

TSA-mediated HIF-1α accumulation is also associated with the activation of Erk1/2 and Akt [16]. Because HIF-1α expression can be induced *via* the production of H_2_O_2_ [34], reactive oxygen or nitrogen species may be required for HIF-1α stabilization [33, 35–38]. However, it is necessary to further define various signaling pathways that are associated with the induction of TSA resistance via HIF-1α.

A previous report showed that TSA is associated with the regulation of P-glycoprotein in drug-resistant cells [39]. p21WAF1 acts as a resistance factor to the combination therapy of TSA and interferon alpha (IFNα) [26]. Overexpression of GRP78/BiP, an antiapoptotic chaperone of the endoplasmic reticulum, reduces HDAC inhibitor-induced apoptosis in cancer cells [25]. Antitumor effects are also inhibited by a decrease in microtubule dynamics or MMP [40]. Therefore, it should not be ruled out the possibility that other pathways are involved in TSA resistance.

In conclusion, our data demonstrate that tumor cell death induced by TSA is inhibited by HIF-1α acetylation, its nuclear translocation and binding to the HRE of the VEGF promoter. Our results suggest that the induction of drug resistance could be concomitant with the anticancer effects of TSA under normoxic conditions, providing additional evidence that the combined anticancer therapy regimen should be clinically beneficial to prevent drug resistance in populations at high risk of tumor relapse.

## MATERIALS AND METHODS

### Reagents

Trichostatin A (TSA) and MTT [3(4,5-dimethyl-thiazol-2-yl)-2,5-diphenyl tetrazolium bromide] were purchased from the Sigma-Aldrich (St. Louis, MO, USA). Antibodies which are reactive with tubulin (T4026) and actin (A2066) were purchased from the Sigma-Aldrich (St. Louis, MO, USA). Antibodies which are reactive with acetylated-lysine (9441S), histone 4 (2592S) and normal rabbit immunoglobulin G (IgG) control (2729S) were purchased from Cell Signaling Technology (Beverly, MA, USA). Antibodies which are reactive with histone 4 (05–499), acetylated-histone 3 (06–599), acetylated-histone 4 (06–866) and normal mouse immunoglobulin G (IgG) control (12–371) were obtained from Milipore (Billerica, MA, USA). Antibodies which are reactive with hemagglutinin A (HA, MAB060) were purchased from R&D Systems, Inc. (Minneapolis, MN, USA). Antibodies which are reactive with HIF-1α (sc-10790), GFP (sc-9996), VEGF (sc-152), Lamin B (sc-6217) and glutathione-S-transferse (GST) (sc-138) were obtained from Santa Cruz Biotechnology, Inc. (Santa Cruz, CA, USA). Antibodies which are specific to acetylated HIF-1α (K674) were kindly provided by professor Jong-Wan Park, College of Medicine, Seoul National University (Seoul, Korea). Luciferase assay system with reporter lysis buffer was purchased from Promega (Madison, WI, USA). Except where indicated, all other materials were obtained from the Sigma-Aldrich (St. Louis, MO, USA).

### Cell culture

HeLa human cervix adenocarcinoma cells, MCF7 human breast adenocarcinoma cells, HCT116 human colorectal carcinoma cells, HepG2 human hepatocellular carcinoma cells and HEK293T human embryonic kidney cells were obtained from the Korea Research Institute of Bioscience and Biotechnology (KRIBB) cell bank (Daejeon, Korea). Cells were maintained and cultured in Dulbecco's modified Eagle's medium (GIBCO BRL, Gaithersburg, MD, USA) supplemented with 10% fetal bovine serum (Hyclone, Kansas City, MO, USA), 2 mM L-glutamine, 100 units/ml penicillin, and 100 units/ml streptomycin (GIBCO BRL, Gaithersburg, MD, USA). No mycoplasma contamination was regularly tested by using mycoplasma detection kit (Thermo Fisher Scientific, Waltham, MA, USA) every other month.

### Plasmids and transient transfection

HRE reporter plasmid 5× VEGF-HRE-pSV40min was kindly provided from Dr. Dong-Soo Im, Korea Research Institute of Bioscience and Biotechnology (KRIBB, Daejeon, Korea). HA-HIF-1α and HA-HIF-1α (K674R) mutant plasmis were kindly provided by professor Jong-Wan Park, College of Medicine, Seoul National University (Seoul, Korea). pEGFP-C1-HIF-1α, pMXs-RFP1 and pSG5 plasmids were kindly provided from professors Mi-Ock Lee, College of Pharmacy, Seoul National University (Seoul, Korea), Jeong-Soo You, School of Medicine, Konkuk University (Seoul, Korea) and Soo-Jong Um, College of Bioscience and Biotechnology, Sejong University (Seoul, Korea), respectively. pSG5-HIF-1α plasmid was generated by subcloning HIF-1α sequences derived from pEGFP-C1-HIF-1α plasmid into the sites of EcoRI and BamHI of pSG5. To express the specific target molecules, the amount of each plasmid in the range of 0.3 ~ 0.5 μg was used for the transfection of each cell line in 35 mm^2^ culture dishes using Lipofectamine 2000 (Invitrogen, Carlsbad, CA, USA). To determine whether GFP-HIF-1α was tanslocated into nucleus by TSA treatment, cells were transfected with pEGFP-C1-HIF-1α plasmids or co-transfected with pEGFP-C1-HIF-1α and pMXs-RFP1 plasmids. Then, over 1,000 single GFP-positive or dual GFP- and RFP-positive cells were respectively counted under fluorescence microscope (Nikon Instruments Korea Co. Ltd, Seoul, Korea)

### MTT assay

We quantified cell survival using colorimetric assay described for measuring intracellular succinate dehydrogenase content with MTT [41]. Confluent cells were cultured with various concentrations of each reagent for 48 h. Cells were then incubated with 50 μg/ml of MTT at 37°C for 2 h. Formazan formed were dissolved in dimethylsulfoxide (DMSO). Optical density (OD) was read at 595 nm.

### Hypoxia response element (HRE) reporter assay

Active transcription factor HIF-1α recognizes and binds to the hypoxia-response elements (HRE; 5′-A/GCGTG-3′) in hypoxia-inducible promoters [42]. HRE reporter plasmid 5× VEGF-HRE-pSV40min was generated by cloning five tandem couples of HRE derived from the human VEGF promoter into the BglII site of pGL3 [43]. To measure the activity of VEGF transcription, confluent HeLa cells were transfected with VEGF-HRE-pSV40min plasmid using Lipofectamine 2000 (Invitrogen, Carlsbad, CA, USA) [44]. At the same time, cells were co-transfected with pcDNA-lacZ for monitoring transfection efficiency by β-galactosidase assay [45]. Luciferase activity was measured by using luciferase assay system with reporter lysis buffer (Promega, Madison, WI, USA). Luminescence was measured using luminometer (Berthold Technologies, Oak Ridge, TN, USA). VEGF-HRE activity was normalized to this control β-galactosidase activity. Fold changes in luciferase units of experimental group were also normalized to the control group [45–47].

### Purification of nuclear fraction

Cells were harvested and suspended with buffer A (25 mM Tris-Cl (pH 8.0) 10 mM KCl 1 mM DTT 0.5 mM PMSF) followed by the incubation on ice for 15 min. Cell suspension was treated with 10% NP-40 to the final concentration of 0.75% NP-40, mixed with vortexing for 30 sec and centrifuged at 1,400 × g for 1 min. Then, pellet was separated from supernatant. Nuclear fraction was obtained by the lysis of pellet in buffer C (50 mM Tris-Cl (pH 8.0) 400 mM NaCl 1 mM DTT 1 mM PMSF), the centrifugation at 15,000 × g for 30 min and the collection of supernatant. Cytoplasmic fraction was obtained by the centrifugation of supernatant at 1,500 × g for 15 min and the collection of its supernatant.

### Chromatin immunoprecipitation (ChIP) assay

ChIP assay was performed as describied previously [48, 49]. Cells were crosslinked with final concentration 1% formaldehyde for 10 min at room temperature. Then 125 mM glycine was added to quench unreacted formaldehyde. Cells were gathered and sonicated to make DNA fragments with a size range of 200 ~ 1000 bp. Cell extracts were immune-precipitated using anti-GST (Santa Cruz, CA, USA) and rabbit IgG control antibodies (Abcam, Cambridge, UK) for all ChIP experiments. PCR analysis were performed by using primer sets spanning the HIF-1α binding site on hVEGF-A gene promoter. Primer sequences are 5′-aac aag ggc ctc tgt ctg c-3′ (–1,037 ~ –1,019 bp) and 5′-ggg gag aag aat ttg gca cc-3′ (–888 ~ –869 bp) including VEGF-HRE.

### Immunoprecipitation

Immunoprecipitation was performed to detect proteins with acetylated lysine including HIF-1α as below. Cell lysates were incubated with rabbit polyclonal anti-acetylated lysine antibodies (Cell Signaling Technology, Beverly, MA, USA) for 2 h. Immunoprecipitates were collected by means of protein A/G PLUS agarose beads (Santa Cruz, CA, USA). Proteins with acetylated lysine were released from the beads by addition of 1× sample buffer and boiling for 5 min. HIF-1α with acetylated lysine was then detected by western blot analysis using an anti-HIF-1α antibody and enhanced chemiluminescence (ECL) (Pierce, Rockford, IL, USA).

### Reverse transcription polymerase chain reaction (RT-PCR)

Total RNA was extracted from HeLa cells using TRIZOL reagent (Invitrogen, Carlsbad, CA, USA). cDNA was synthesized from 1μg of total RNA using oligo-dT_18_ primers and reverse transcriptase in a total volume of 21 μl (Bioneer, Daejeon, Korea). For standard PCR, 1 μL of the first-strand cDNA product as a template and 10 pmol of specific primers were used for PCR amplification with Taq DNA polymerase (Cosmo Genetech, Seoul, Korea). PCR amplification was performed using oligonucleotides specific for target genes, HIF-1α, VEGF, VEGF-A, VEGF-B, VEGF-C, VEGF-D, and β-actin (Table 1). PCR products were detected by 1.2% agarose gel electrophoresis with a marker of 100 bp ladder.

**Table 1:** Sequences of oligonucleotides specific for RT-PCR to target genes

Target gene	Forward primer	Reverse primer
HIF-1α	5′-ctc aaa gtc gga cag cct ca-3′	5′-gat tgc ccc agc agt cta ca-3′
VEGF	5′-tga cag gga aga gga gga ga-3′	5′-tgg ttt caa tgg tgt gag ga-3′
VEGF-A	5′-ctt gcc ttg ctg ctc tac ct	5′-gca gta gct gcg ctg ata ga-3′
VEGF-B	5′-tgt ata ctc gcg cta cct gc	5′-atc tgc atc cgg act tgg tg-3′
VEGF-C	5′-gac ctg ccc cac caa tta ca	5′-tcc agc tcc ttg ttt ggt ct
VEGF-D	5′-gaa cac cag cac ctc gta ca	5′-aca gac aca ctc gca acg at
β-actin	5′-gtc acc aac tgg gac gac at-3′	5′-gca cag cct gga tag caa cg-3′

### Realtime quantitative(Q)-PCR analysis

To perform real-time quantitative(Q)-PCR, total cellular RNA (1 μg) was reverse transcribed into cDNA as described in RT-PCR. Real-time Q-PCR was performed using the CFX96 Touch™ Real-Time PCR Detection System (Bio-Rad laboratories, Hercules, CA, USA). The RT reaction product (10 ng) was amplified with Thunderbird SYBR qPCR mix (TOYOBO Co. Ltd., Osaka, Japan) using primers specific for target genes, HIF-1α primers (forward; 5′-gat gat gac ttc cag tta cg-3′ and reverse; 5′-tgg tag tgg tgg cat tag-3′), VEGF-A primers (forward; 5′-gga gtc caa cat cac cat-3′ and reverse; 5′-tgt ctt gct cta tct ttc ttt g-3′) and β-actin primers (forward; 5′-gcc agg tca tca cca ttg-3′, reverse; 5′-gtt gaa ggt agt ttc gtg gat-3′). Samples were heated to 95°C for 1 min and amplified for 40 cycles (95°C for 10 s, 55°C for 10 s and 72°C for 30 s) followed by a final extension step of 72°C for 10 min. β-actin was used as an internal control. Relative quantification of each mRNA was analyzed by the comparative threshold cycle (CT) method and normalized to β-actin expression using Bio-Rad CFX Manager™ Software.

### Western blot analysis

Western blotting was performed using a standard protocol. Cells were lysed in ice-cold lysis buffer containing 0.5% Nonidet P-40 (vol/vol) in 20 mM Tris-HCl (pH 8.3); 150 mM NaCl; protease inhibitors (2 mg/ml aprotinin, pepstatin, and chymostatin; 1 mg/ml leupeptin and pepstatin; 1 mM phenylmethyl sulfonyl fluoride (PMSF); and 1 mM Na_4_VO_3_. Lysates were incubated for 1 h on ice before centrifugation at 13,000 × g for 10 min at 4°C. Protein concentration in each supernatant was measured using a SMART™ BCA protein assay kit (iNtRON Biotech. Inc., Seoul, Republic of Korea). Proteins were denatured by boiling for 5 min in sodium dodecyl sulfate(SDS) sample buffer. Proteins were separated by 12% SDS-polyacrylamide gel electrophoresis(SDS-PAGE), and transferred to nitrocellulose membranes by electro-blotting. Following transfer, equal loading of protein was verified by Ponceau staining. The membranes were blocked with 5% skim milk in Tris-buffered saline with Tween 20 (TBST) (10 mM Tris-HCl, pH 7.6; 150 mM NaCl; 0.5% Tween 20) and incubated with the indicated antibodies. Most antibodies were diluted to 1:000, except anti-actin and anti-tubulin antibodies to 1:5000, anti-histone 3/4 and acetylated histone 3/4 antibodies to 1:3000 with TBST. Bound antibodies were visualized with HRP-conjugated secondary antibodies with the use of enhanced chemiluminescence (ECL) (Pierce, Rockford, IL, USA). Immunoreactive bands were detected using X-ray film.

### Statistical analyses

Experimental differences were examined using ANOVA and Students’ *t*-tests, as appropriate. *P* values of < 0.05 or < 0.01 were considered to indicate significance.

## References

[1] Emmenegger U, Kerbel RS (2010). Cancer: Chemotherapy counteracted. Nature.

[2] Masoud GN, Li W (2015). HIF-1αlpha pathway: role, regulation and intervention for cancer therapy. Acta Pharm Sin B.

[3] Semenza GL, Wang GL (1992). A nuclear factor induced by hypoxia via de novo protein synthesis binds to the human erythropoietin gene enhancer at a site required for transcriptional activation. Mol Cell Biol.

[4] Semenza GL (2003). Targeting HIF-1 for cancer therapy. Nat Rev Cancer.

[5] Wang GL, Semenza GL (1993). General involvement of hypoxia-inducible factor 1 in transcriptional response to hypoxia. Proc Natl Acad Sci U S A.

[6] Birner P, Schindl M, Obermair A, Breitenecker G, Oberhuber G (2001). Expression of hypoxia-inducible factor 1alpha in epithelial ovarian tumors: its impact on prognosis and on response to chemotherapy. Clin Cancer Res.

[7] Zagzag D, Zhong H, Scalzitti JM, Laughner E, Simons JW, Semenza GL (2000). Expression of hypoxia-inducible factor 1alpha in brain tumors: association with angiogenesis, invasion, and progression. Cancer.

[8] Jaakkola P, Mole DR, Tian YM, Wilson MI, Gielbert J, Gaskell SJ, von Kriegsheim A, Hebestreit HF, Mukherji M, Schofield CJ, Maxwell PH, Pugh CW, Ratcliffe PJ (2001). Targeting of HIF-alpha to the von Hippel-Lindau ubiquitylation complex by O2-regulated prolyl hydroxylation. Science.

[9] Ohh M, Park CW, Ivan M, Hoffman MA, Kim TY, Huang LE, Pavletich N, Chau V, Kaelin WG (2000). Ubiquitination of hypoxia-inducible factor requires direct binding to the beta-domain of the von Hippel-Lindau protein. Nat Cell Biol.

[10] Oh JM, Moon EY (2010). Actin-sequestering protein, thymosin beta-4, induces paclitaxel resistance through ROS/HIF-1αlpha stabilization in HeLa human cervical tumor cells. Life Sci.

[11] Oh JM, Ryu YK, Lim JS, Moon EY (2010). Hypoxia Induces Paclitaxel-Resistance through ROS Production. Biomol Ther.

[12] Brown JM (2007). Tumor hypoxia in cancer therapy. Methods Enzymol.

[13] Tsuji N, Kobayashi M, Nagashima K, Wakisaka Y, Koizumi K (1976). A new antifungal antibiotic, trichostatin. J Antibiot (Tokyo).

[14] Yoshida M, Kijima M, Akita M, Beppu T (1990). Potent and specific inhibition of mammalian histone deacetylase both *in vivo* and *in vitro* by trichostatin A. J Biol Chem.

[15] Rasheed WK, Johnstone RW, Prince HM (2007). Histone deacetylase inhibitors in cancer therapy. Expert Opin Investig Drugs.

[16] Huang X, Li Y, Li J, Feng Y, Xu X (2014). Tanshinone IIA dampens the cell proliferation induced by ischemic insult in rat astrocytes via blocking the activation of HIF-1αlpha/SDF-1 signaling. Life Sci.

[17] Geng H, Harvey CT, Pittsenbarger J, Liu Q, Beer TM, Xue C, Qian DZ (2011). HDAC4 protein regulates HIF1alpha protein lysine acetylation and cancer cell response to hypoxia. J Biol Chem.

[18] Mizuno S, Yasuo M, Bogaard HJ, Kraskauskas D, Natarajan R, Voelkel NF (2011). Inhibition of histone deacetylase causes emphysema. Am J Physiol Lung Cell Mol Physiol.

[19] Ninios YP, Sekeri-Pataryas KE, Sourlingas TG (2010). Differential sensitivity of human leukemic cell lines to the histone deacetylase inhibitor, trichostatin A. Leuk Res.

[20] Kang FW, Que L, Wu M, Wang ZL, Sun J (2012). Effects of trichostatin A on HIF-1αlpha and VEGF expression in human tongue squamous cell carcinoma cells *in vitro*. Oncol Rep.

[21] Kim SH, Jeong JW, Park JA, Lee JW, Seo JH, Jung BK, Bae MK, Kim KW (2007). Regulation of the HIF-1αlpha stability by histone deacetylases. Oncol Rep.

[22] Yang QC, Zeng BF, Shi ZM, Dong Y, Jiang ZM, Huang J, Lv YM, Yang CX, Liu YW (2006). Inhibition of hypoxia-induced angiogenesis by trichostatin A via suppression of HIF-1α activity in human osteosarcoma. J Exp Clin Cancer Res.

[23] Yu J, Mi J, Wang Y, Wang A, Tian X (2012). Regulation of radiosensitivity by HDAC inhibitor trichostatin A in the human cervical carcinoma cell line Hela. Eur J Gynaecol Oncol.

[24] Hauswald S, Duque-Afonso J, Wagner MM, Schertl FM, Lubbert M, Peschel C, Keller U, Licht T (2009). Histone deacetylase inhibitors induce a very broad, pleiotropic anticancer drug resistance phenotype in acute myeloid leukemia cells by modulation of multiple ABC transporter genes. Clin Cancer Res.

[25] Baumeister P, Dong D, Fu Y, Lee AS (2009). Transcriptional induction of GRP78/BiP by histone deacetylase inhibitors and resistance to histone deacetylase inhibitor-induced apoptosis. Mol Cancer Ther.

[26] Kuljaca S, Liu T, Tee AE, Haber M, Norris MD, Dwarte T, Marshall GM (2007). Enhancing the anti-angiogenic action of histone deacetylase inhibitors. Mol Cancer.

[27] Yang DH, Lee JW, Lee J, Moon EY (2014). Dynamic rearrangement of F-actin is required to maintain the antitumor effect of trichostatin A. PLoS One.

[28] Mottamal M, Zheng S, Huang TL, Wang G (2015). Histone deacetylase inhibitors in clinical studies as templates for new anticancer agents. Molecules.

[29] Azechi T, Kanehira D, Kobayashi T, Sudo R, Nishimura A, Sato F, Wachi H (2013). Trichostatin A, an HDAC class I/II inhibitor, promotes Pi-induced vascular calcification via up-regulation of the expression of alkaline phosphatase. J Atheroscler Thromb.

[30] Vigushin DM, Ali S, Pace PE, Mirsaidi N, Ito K, Adcock I, Coombes RC (2001). Trichostatin A is a histone deacetylase inhibitor with potent antitumor activity against breast cancer *in vivo*. Clin Cancer Res.

[31] Reynoso-Roldan A, Roldan ML, Cancino-Diaz JC, Rodriguez-Martinez S, Cancino-Diaz ME (2012). Vascular endothelial growth factor production is induced by histone deacetylase 1 and suppressed by von Hippel-Lindau protein in HaCaT cells. Clin Invest Med.

[32] Lim JH, Lee YM, Chun YS, Chen J, Kim JE, Park JW (2010). Sirtuin 1 modulates cellular responses to hypoxia by deacetylating hypoxia-inducible factor 1alpha. Mol Cell.

[33] Jeong JW, Bae MK, Ahn MY, Kim SH, Sohn TK, Bae MH, Yoo MA, Song EJ, Lee KJ, Kim KW (2002). Regulation and destabilization of HIF-1αlpha by ARD1-mediated acetylation. Cell.

[34] Gao N, Shen L, Zhang Z, Leonard SS, He H, Zhang XG, Shi X, Jiang BH (2004). Arsenite induces HIF-1αlpha and VEGF through PI3K, Akt and reactive oxygen species in DU145 human prostate carcinoma cells. Mol Cell Biochem.

[35] Brunelle JK, Bell EL, Quesada NM, Vercauteren K, Tiranti V, Zeviani M, Scarpulla RC, Chandel NS (2005). Oxygen sensing requires mitochondrial ROS but not oxidative phosphorylation. Cell Metab.

[36] Guzy RD, Hoyos B, Robin E, Chen H, Liu L, Mansfield KD, Simon MC, Hammerling U, Schumacker PT (2005). Mitochondrial complex III is required for hypoxia-induced ROS production and cellular oxygen sensing. Cell Metab.

[37] Kaelin WG (2005). ROS: really involved in oxygen sensing. Cell Metab.

[38] Mansfield KD, Guzy RD, Pan Y, Young RM, Cash TP, Schumacker PT, Simon MC (2005). Mitochondrial dysfunction resulting from loss of cytochrome c impairs cellular oxygen sensing and hypoxic HIF-alpha activation. Cell Metab.

[39] Castro-Galache MD, Ferragut JA, Barbera VM, Martin-Orozco E, Gonzalez-Ros JM, Garcia-Morales P, Saceda M (2003). Susceptibility of multidrug resistance tumor cells to apoptosis induction by histone deacetylase inhibitors. Int J Cancer.

[40] Lee JW, Park S, Kim SY, Um SH, Moon EY (2016). Curcumin hampers the antitumor effect of vinblastine via the inhibition of microtubule dynamics and mitochondrial membrane potential in HeLa cervical cancer cells. Phytomedicine.

[41] Denizot F, Lang R (1986). Rapid colorimetric assay for cell growth and survival. Modifications to the tetrazolium dye procedure giving improved sensitivity and reliability. J Immunol Methods.

[42] Obach M, Navarro-Sabate A, Caro J, Kong X, Duran J, Gomez M, Perales JC, Ventura F, Rosa JL, Bartrons R (2004). 6-Phosphofructo-2-kinase (pfkfb3) gene promoter contains hypoxia-inducible factor-1 binding sites necessary for transactivation in response to hypoxia. J Biol Chem.

[43] Cho WK, Seong YR, Lee YH, Kim MJ, Hwang KS, Yoo J, Choi S, Jung CR, Im DS (2004). Oncolytic effects of adenovirus mutant capable of replicating in hypoxic and normoxic regions of solid tumor. Mol Ther.

[44] Oh JM, Ryoo IJ, Yang Y, Kim HS, Yang KH, Moon EY (2008). Hypoxia-inducible transcription factor (HIF)-1 alpha stabilization by actin-sequestering protein, thymosin beta-4 (TB4) in Hela cervical tumor cells. Cancer Lett.

[45] Lee GH, Lee MH, Yoon YD, Kang JS, Pyo S, Moon EY (2012). Protein kinase C stimulates human B cell activating factor gene expression through reactive oxygen species-dependent c-Fos in THP-1 pro-monocytic cells. Cytokine.

[46] Lee GH, Lee J, Lee JW, Choi WS, Moon EY (2013). B cell activating factor-dependent expression of vascular endothelial growth factor in MH7A human synoviocytes stimulated with tumor necrosis factor-alpha. Int Immunopharmacol.

[47] Ryu YK, Lee JW, Moon EY (2015). Thymosin Beta-4, Actin-Sequestering Protein Regulates Vascular Endothelial Growth Factor Expression via Hypoxia-Inducible Nitric Oxide Production in HeLa Cervical Cancer Cells. Biomol Ther (Seoul).

[48] Cawley S, Bekiranov S, Ng HH, Kapranov P, Sekinger EA, Kampa D, Piccolboni A, Sementchenko V, Cheng J, Williams AJ, Wheeler R, Wong B, Drenkow J (2004). Unbiased mapping of transcription factor binding sites along human chromosomes 21 and 22 points to widespread regulation of noncoding RNAs. Cell.

[49] Spencer VA, Sun JM, Li L, Davie JR (2003). Chromatin immunoprecipitation: a tool for studying histone acetylation and transcription factor binding. Methods.

